# Wnt/Notch Crosstalk Promotes Epithelial–Mesenchymal Transition During *Chlamydia trachomatis* Infection Under IFN-γ Treatment

**DOI:** 10.3390/microorganisms14092074

**Published:** 2026-09-17

**Authors:** Yewei Yang, Chunxia Fang, Hongrong Wu, Lili Chen, Yating Wen, Zhongyu Li

**Affiliations:** Hunan Provincial Key Laboratory for Special Pathogens Prevention and Control, Hengyang Medical School, Institute of Pathogenic Biology, School of Nursing, University of South China, Hengyang 421001, China; viktoryang@163.com (Y.Y.); fcx1195329675@163.com (C.F.); wuhongrong@usc.edu.cn (H.W.); chlili720612@163.com (L.C.); wenyating@usc.edu.cn (Y.W.)

**Keywords:** *Chlamydia trachomatis*, epithelial–mesenchymal transition, Wnt/β-catenin signaling pathway, Notch signaling pathway, infection

## Abstract

*Chlamydia trachomatis* (Ct) infection is a major cause of infertility, primarily through inducing tubal fibrosis. Epithelial–mesenchymal transition (EMT) plays a central role in this fibrotic process. However, the molecular mechanisms by which Ct infection triggers EMT are not fully understood. IFN-γ is a major component of the host immune pressure acting on infected epithelial cells during Ct infection. To mimic this immune environment in vitro and investigate how host signaling responses to Ct infection influence EMT, we maintained Ct-infected HeLa cells under continuous IFN-γ treatment and examined Wnt/β-catenin and Notch signaling. Under this condition, both pathways were activated and exhibited functional crosstalk potentially involving JAG1. Inhibition of either pathway attenuated EMT-associated phenotypes and was associated with reduced infectious progeny production. These findings support a role for Wnt/β-catenin–Notch crosstalk in promoting EMT in Ct-infected cells under continuous IFN-γ treatment.

## 1. Introduction

*Chlamydia trachomatis* (Ct), an obligate intracellular bacterium, is a leading cause of sexually transmitted bacterial infections worldwide. The majority of infected women remain asymptomatic [1], leaving infections undetected and untreated, thereby facilitating the establishment of infection in the genital tract. If left untreated, ascending infection can involve the upper reproductive tract, causing endometritis, salpingitis, and pelvic inflammatory disease (PID) [2,3]. with serious sequelae including tubal occlusion, infertility, and ectopic pregnancy [4].

A particularly important consequence of upper genital tract Ct infection is tubal scarring and fibrosis, and accumulating experimental evidence suggests that epithelial–mesenchymal transition (EMT) may contribute to this pathological remodeling [5,6,7]. During EMT, epithelial cells progressively lose apical–basal polarity and intercellular adhesion and acquire mesenchymal features associated with increased cellular motility [8]. This process is commonly accompanied by reduced expression of epithelial markers such as E-cadherin and increased expression of mesenchymal markers, including vimentin and N-cadherin [9]. In relation to the fibrotic remodeling associated with Ct infection, recent studies have identified host signaling programs that may link epithelial responses to subsequent fibrotic remodeling. Ct-induced activation of YAP-dependent transcription and profibrotic signaling has been demonstrated in endocervical epithelial cells [10]. Integrated miRNA–mRNA profiling of endometrial tissues from women with upper genital tract Ct infection further revealed reduced expression of miRNAs that normally suppress TGF-β-associated EMT pathways [11]. Moreover, Ct-infected endocervical epithelial cells stimulated type I collagen production by neighboring fibroblasts through paracrine signaling, an effect attenuated by YAP knockdown in the infected epithelial cells [12]. These findings implicate epithelial signaling and EMT-associated pathways in fibrotic remodeling associated with Ct infection, but the signaling mechanisms coordinating these responses under conditions of infection remain incompletely defined [7].

Among the signaling networks potentially coordinating EMT during Ct infection, the Wnt/β-catenin and Notch pathways are of particular interest because both regulate epithelial plasticity and pathological tissue remodeling [13,14]. Experimental evidence indicates that Ct can engage host Wnt/β-catenin signaling in epithelial cells; Ct-induced FGD5-AS1 expression promotes β-catenin accumulation and nuclear translocation and contributes to host-cell survival [15]. Notch signaling is also a recognized regulator of EMT, and studies in fibrotic models have demonstrated functional interactions between Wnt/β-catenin and the JAG1/Notch axis, suggesting a potential mechanism through which these pathways may coordinately regulate epithelial and profibrotic responses [16]. However, whether Wnt/β-catenin and Notch signaling are functionally coupled during Ct infection, and whether such coupling contributes to EMT, remains unclear.

Ct infection elicits IFN-γ secretion from host immune cells. In vivo, IFN-γ upregulates indoleamine 2,3-dioxygenase (IDO), which depletes tryptophan and places immune pressure on intracellular Ct. Continuous IFN-γ treatment is widely used in vitro to research host–pathogen interactions under sustained immune pressure. Our group previously adopted this approach in Ct-infected HeLa cells using 75 U/mL IFN-γ [17]. In the present study, we compared uninfected and Ct-infected HeLa cells maintained under continuous IFN-γ treatment to examine changes in Wnt/β-catenin and Notch signaling and EMT. We then investigated the functional interaction between these pathways, the potential involvement of JAG1, and their effects on EMT and infectious progeny production. Our findings identify functional Wnt/β-catenin–Notch crosstalk, potentially involving JAG1, that promotes EMT in Ct-infected cells under this condition.

## 2. Materials and Methods

### 2.1. Cell Culture

HeLa 229 cells were obtained from the American Type Culture Collection (RRID: CVCL_1276, ATCC CCL-2) and were cultured in Dulbecco’s Modified Eagle Medium (DMEM; Gibco, Waltham, MA, USA) supplemented with 10% fetal bovine serum (FBS; Gibco) at 37 °C in a 5% CO_2_ incubator. Upon reaching approximately 75% confluence, cells were seeded into 6-well or 24-well plates and incubated overnight at 37 °C in 5% CO_2_ prior to experimental use. Ct serovar E standard strain (ATCC VR-348B; GenBank: JX559522, Manassas, VA, USA) was obtained from the American Type Culture Collection (ATCC).

### 2.2. Ct Infection Under Continuous IFN-γ Treatment

The continuous IFN-γ treatment regimen was based on a protocol previously used by our group [17]. Cells were incubated with 30 μg/mL DEAE-dextran for 10 min, and the solution was then removed. After washing with PBS, either the infection solution (MOI = 1) or culture medium alone for mock controls was added, and plates were centrifuged at 300× *g* for 60 min to assist infection. The medium in both the infection and mock groups was then replaced with fresh medium (380 μL per well) containing recombinant human IFN-γ (75 U/mL; Solarbio, Beijing, China), and IFN-γ exposure was maintained continuously until sample collection at 12, 24, or 40 h post-infection. All experiments involving IWP2 or DAPT were performed under the same IFN-γ-induced infection regimen. IWP2 and DAPT were dissolved in DMSO and tested at final concentrations of 10 and 20 μM in the dose–response experiments. Based on their previously reported use in HeLa-cell systems [15,18].

### 2.3. Immunofluorescence Analysis

HeLa cells were washed three times with PBS, then fixed in 4% paraformaldehyde at 37 °C for 25 min. Next, the cells were treated with 0.25% Triton X-100 for 20 min at 37 °C to make them permeable. After that, they were blocked with DMEM containing 10% FBS for 1 h at 37 °C. Following two more PBS washes, rabbit polyclonal antibodies against Ct serovar E were diluted 1:500 in DMEM and added to the cells for overnight incubation at 4 °C. The next day, Cy-2 secondary antibodies (Proteintech, Chicago, IL, USA) and DAPI staining (Sigma-Aldrich, Munich, ND, USA) were diluted in DMEM to avoid light and incubated for 1 h at 37 °C. At the end of the procedure, inclusions were checked and photographed under an inverted fluorescence microscope.

### 2.4. Western Blot Analysis

The samples were treated on ice for 30 min with 100 μL RIPA (Solarbio, Beijing, China) cell lysate containing 1 mM PMSF (Solarbio, Beijing, China). The cells were equally scraped, and the lysate was collected in an EP tube. The supernatant was obtained after 10 min of centrifugation at 4000× *g*, and the protein concentration was measured using the BCA method (Beyotime Biotech, Nanjing, China). Equal amounts of total protein (20 μg per lane) were mixed with SDS loading buffer, denatured at 95 °C for 5 min, and separated by 10% SDS-PAGE gel electrophoresis. Proteins were then transferred onto 0.22 μm PVDF membranes (Millipore, Billerica, MA, USA). The membrane was then blocked using 5% non-fat milk solution. After blocking, it was incubated overnight with primary antibodies targeting Vimentin, E-cadherin, N-cadherin, Notch-1, β-catenin, β-actin, and JAG1 (all from CST, Danvers, MA, USA). After the membrane was washed, a secondary antibody (HRP-linked goat anti-rabbit IgG, Proteintech) was added and allowed to incubate. Next, the ECL detection solution (Omni-ECL™ Femto Light Chemiluminescence Kit, Epizyme, Shanghai, China) was prepared and gently applied to the PVDF membrane in the dark. The signal was then captured using the G:Box Chemi XX9 imaging system (Syngene, Cambridge, UK). Background-subtracted band intensities were quantified with Quantity One, and each target was normalized to β-actin from the same sample. Data represents 3 independent biological experiments.

### 2.5. Cell Counting Kit-8 (CCK-8) Assay

HeLa cells were seeded into 96-well plates at 5000 cells per well in 100 μL of DMEM supplemented with 10% fetal bovine serum and cultured at 37 °C in a humidified incubator with 5% CO_2_. After 24 h of incubation, cells were either infected with Ct at a multiplicity of infection (MOI) of 1 or left uninfected as mock controls. All wells were treated with recombinant human IFN-γ at a final concentration of 75 U/mL. At 12 and 24 h after infection, the medium was replaced with 100 μL of fresh DMEM containing 10% CCK-8 reagent (DoJinDo Laboratories, Kumamoto, Japan), followed by a 1-h incubation at 37 °C. Absorbance at 450 nm was then recorded using a microplate reader (Thermo Fisher Scientific, San Diego, CA, USA). Cell viability was expressed as a percentage relative to untreated control cells, which were set at 100%.

### 2.6. Wound Healing Assay

To assess the migratory ability of HeLa cells, a wound healing assay was performed. Cells were first plated in six-well plates and grown until reaching approximately 90% confluence. A straight scratch was gently made across the cell layer using a 200 μL pipette tip. Each well was washed twice with PBS, followed by treatment with 30 μg/mL DEAE-dextran (Diethylaminoethyl dextran gel) for 10 min. The DEAE solution was then removed, and the cells were washed again with PBS. The bacterial suspension (MOI = 1) was added immediately after, and the plates were centrifuged at 300× *g* for 1 h to promote infection. For the mock group, the same DEAE pretreatment was applied, but no bacteria were added. After washing with PBS, culture medium was added, followed by centrifugation at 300× *g* for 60 min, and all groups were treated with human IFN-γ at a concentration of 75 U/mL. At the specified time points after scratching, cells in each well were observed and photographed using an inverted microscope. The distance between the edges of the wound was measured with ImageJ 1.54p software (NIH, Bethesda, MD, USA).

### 2.7. Quantitative Real-Time PCR

Total RNA was extracted using TRIzol reagent (Tiangen, Beijing, China) according to the manufacturer’s instructions. RNA concentration and purity were assessed by spectrophotometry, and samples with an A260/A280 ratio of 1.8–2.0 were used. cDNA was synthesized from 1 μg of total RNA using a reverse transcription kit (Tiangen, Beijing, China). RT-qPCR was performed using SuperReal PreMix Plus (Tiangen, Beijing, China) on a LightCycler 96 system (Roche, Basel, Switzerland) in a 20 μL reaction volume. The cycling conditions were 95 °C for 15 min, followed by 40 cycles of 95 °C for 10 s, 60 °C for 20 s, and 72 °C for 30 s, followed by melting-curve analysis. Relative expression levels of NOTCH1 and HES1 were calculated using the 2^−ΔΔCt^ method with GAPDH as the reference gene. Each sample was analyzed in triplicate, and three independent biological experiments were performed. Primer sequences are listed in Table 1.

### 2.8. RNA Interference

Sangon Biotech (Shanghai, China) provided the siRNA targeting JAG1 (si-JAG1) and a non-targeting control (si-NC). HeLa cells were plated in 6-well plates, and when cell confluence reached about 60% to 70%, they were transfected with 50 nM si-JAG1 or si-NC using Lipofectamine 3000 (Invitrogen, Carlsbad, CA, USA). After an 8-h transfection period, the medium was replaced with fresh DMEM containing 10% fetal bovine serum. The siRNA sequence targeting JAG1 was 5′-GCAACACCTTCAACCTCAA-3′.

### 2.9. Infectious Progeny Titration Assay

HeLa cells were cultured in 24-well plates and infected with Ct serovar E at an MOI of 1 under continuous IFN-γ treatment (75 U/mL), as described above. The Ct inoculum used for primary infection had an infectious titer of 3.240 × 10^7^ IFU/mL. Selected cultures were supplemented with IWP2, DAPT, or DMSO. Following 40 h of infection, the treatment medium was removed, and the monolayers were washed twice with PBS. Cells were then lysed with glass beads in fresh medium without IFN-γ or inhibitors. After centrifugation at 1000 rpm for 5 min, the clarified lysates were used to infect fresh untreated HeLa monolayers under standard culture conditions without IFN-γ, IWP2, or DAPT for 40 h. Cells were then fixed and stained with an anti-Ct primary antibody and fluorescent secondary antibody. Inclusions were counted in five randomly selected fields per sample, and these counts were used to calculate the infectious progeny titer, expressed as IFU/mL.

### 2.10. Bioinformatics Analysis

The RNA-sequencing data from Ct-infected cells maintained under the same continuous IFN-γ treatment regimen were obtained from our previously published work. The raw data files have been deposited in the NCBI Sequence Read Archive (SRA) under BioProject accession number PRJNA1036572 (https://www.ncbi.nlm.nih.gov/bioproject/PRJNA1036572, accessed on 5 March 2025). No new sequencing was performed for the present study. These transcriptomic profiles were re-analyzed using the LC-Bio Cloud Platform (https://www.omicstudio.cn/tool, accessed on 5 March 2025). Differentially expressed genes (DEGs) were defined by an absolute log2 fold change (|log2FC|) > 1 and an adjusted *p*-value < 0.05. Gene Ontology (GO) enrichment analysis was performed on these DEGs to categorize enriched terms into biological processes (BP), cellular components (CC), and molecular functions (MF). Enrichment significance was evaluated using Fisher’s exact test, with a *p* < 0.05 considered statistically significant.

### 2.11. Statistical Analysis

Statistical analyses were performed using SPSS v.18.0 and GraphPad Prism v.8. Data are presented as the mean ± s.d. from at least three independent biological replicates; exact *n* values are provided in the figure legends. Normality and homogeneity of variance were assessed using Shapiro–Wilk and Levene’s tests, respectively. Two-group comparisons used two-sided unpaired Student’s *t*-tests, whereas multiple-group comparisons used one-way ANOVA followed by Dunnett’s test. *p* < 0.05 was considered statistically significant.

## 3. Results

### 3.1. Wnt/β-Catenin and Notch Signaling Are Activated in Ct-Infected Cells Under Continuous IFN-γ Treatment

**Figure 1:**
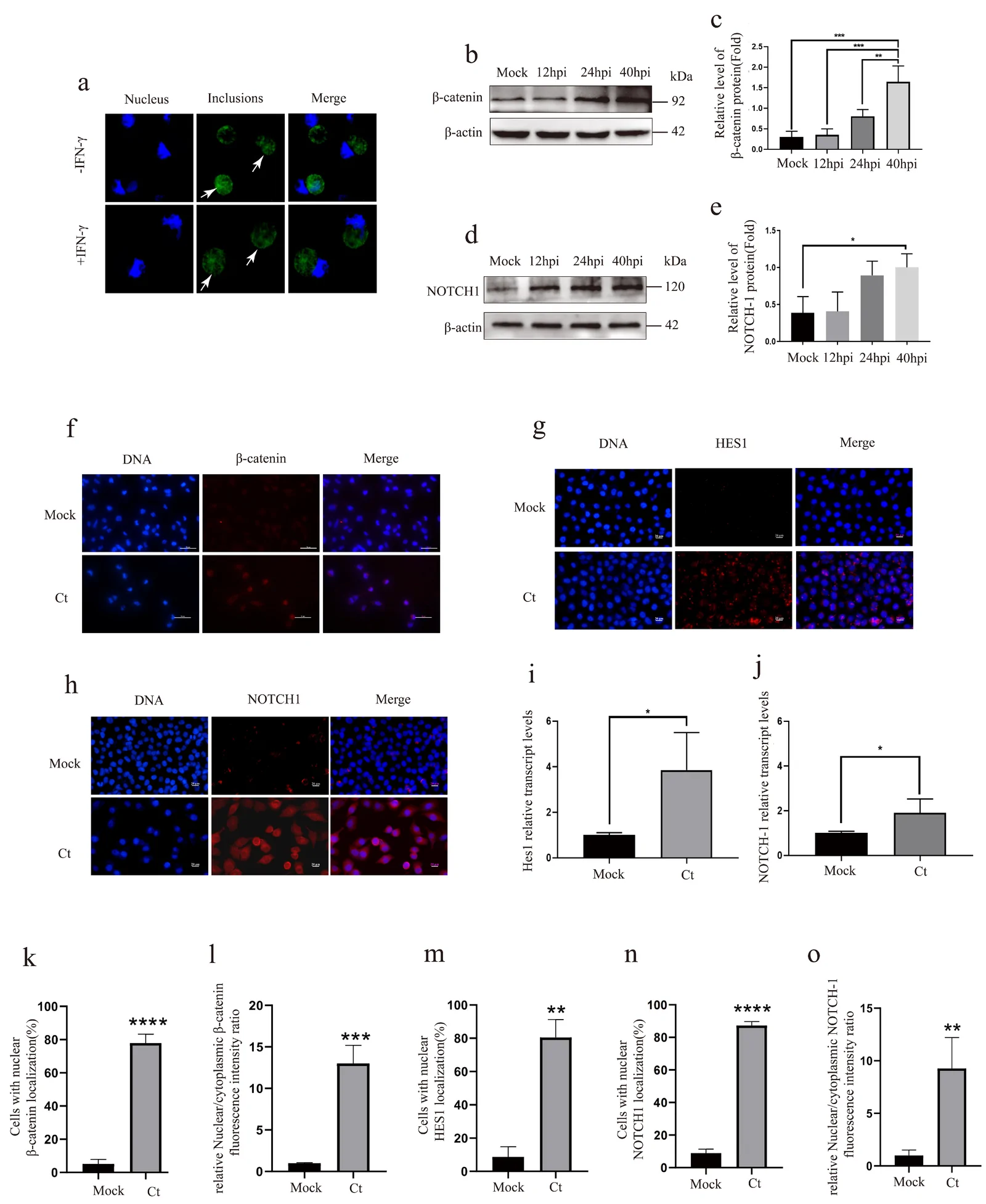
**Wnt/β-Catenin and Notch signaling pathways are activated in** **Ct-infected cells maintained with IFN-γ**. (**a**) Immunofluorescence images showing inclusion morphology in Ct-infected HeLa cells cultured with or without IFN-γ for 24 h. Nuclei were stained with DAPI (blue), and chlamydial inclusions were visualized with Cy2 (green). White arrows indicate chlamydial inclusions. (**b**–**e**) Western blot analysis and densitometric quantification of β-catenin and Notch-1, respectively, at the indicated time points. Protein levels were normalized to β-actin, quantitative data are presented as the mean ± SD from 3 independent biological experiments. Statistical significance was assessed using one-way ANOVA. ** *p* < 0.005; *** *p* < 0.0005, **** *p* < 0.00005. (**f**–**h**) Immunofluorescence images showing the subcellular localization of β-catenin, Hes1, and Notch-1, respectively, at 24 h post-infection, Scale bar in (**f**) = 50 μm; scale bars in (**g**,**h**) = 20 μm. (**i**,**j**) Relative mRNA levels of NOTCH1 and HES1 measured by RT-qPCR. Quantitative data are presented as the mean ± SD from 3 independent biological experiments. Statistical significance was assessed using *t* tests. ** *p* < 0.005; *** *p* < 0.0005, **** *p* < 0.00005. (**k**,**l**) Quantification of β-catenin nuclear localization in mock and Ct-infected cells at 24 h post-infection. (**k**) Percentage of cells exhibiting nuclear β-catenin localization. (**l**) Nuclear-to-cytoplasmic fluorescence intensity ratio of β-catenin. (**m**–**o**) Quantification of nuclear HES1 and NOTCH1 localization in mock- and Ct-infected cells at 24 h post-infection. (**m**) Percentage of cells exhibiting nuclear HES1 localization. (**n**) Percentage of cells exhibiting nuclear NOTCH1 localization. (**o**) Nuclear-to-cytoplasmic fluorescence intensity ratio of NOTCH1. Data is presented as mean ± SD from at least three independent biological replicates. Statistical significance was assessed by unpaired two-tailed Student’s *t*-test. * *p* < 0.05, ** *p* < 0.005, *** *p* < 0.0005.

To investigate host signaling under continuous IFN-γ treatment, we compared uninfected HeLa cells (mock) with Ct-infected HeLa cells, with both groups maintained in medium containing 75 U/mL IFN-γ [17]. Given the established roles of Wnt/β-catenin and Notch signaling in epithelial plasticity and EMT, we examined whether these pathways were activated under this defined condition. At 24 h post-infection, representative IFA images documented Ct inclusions in HeLa cells (Figure 1a). Immunoblot analysis at 12, 24, and 40 h post-infection showed a progressive increase in β-catenin abundance (Figure 1b,c). We further investigated the subcellular localization of β-catenin by IFA in HeLa cells at 24 h post-infection. β-catenin was predominantly localized to the nucleus in infected cells, whereas it remained largely cytosolic in the mock group (Figure 1f). Quantitative analysis further showed an increased percentage of cells with nuclear β-catenin localization and a higher nuclear-to-cytoplasmic fluorescence intensity ratio in Ct-infected group (Figure 1k,l). These results indicate activation of Wnt/β-catenin signaling in Ct-infected cells under continuous IFN-γ treatment.

Given that both Wnt/β-catenin and Notch signaling have been implicated in EMT regulation, we next examined whether Notch signaling is similarly activated during infection. Notch-1 expression was analyzed by Western blot in the Ct and mock groups. Notch-1 was significantly elevated compared to the mock group (*p* < 0.05) (Figure 1d,e). To further confirm this activation, we analyzed the expression of Notch-1 and its downstream effector Hes1 by IFA. Both proteins showed higher nuclear expression in infected cells compared to the mock group (Figure 1g,h). Quantitative analysis showed increased percentages of cells with nuclear Hes1 and Notch-1 localization, together with a higher nuclear-to-cytoplasmic Notch-1 fluorescence intensity ratio in infected cells (Figure 1m–o). Supporting these findings, qPCR analysis revealed significant increases in Notch-1 and Hes1 mRNA levels (*p* < 0.05) (Figure 1i,j). Collectively, these findings support concurrent activation of Wnt/β-catenin and Notch signaling during Ct infection under continuous IFN-γ treatment.

### 3.2. Wnt/β-Catenin and Notch Signaling Exhibit Functional Crosstalk

**Figure 2:**
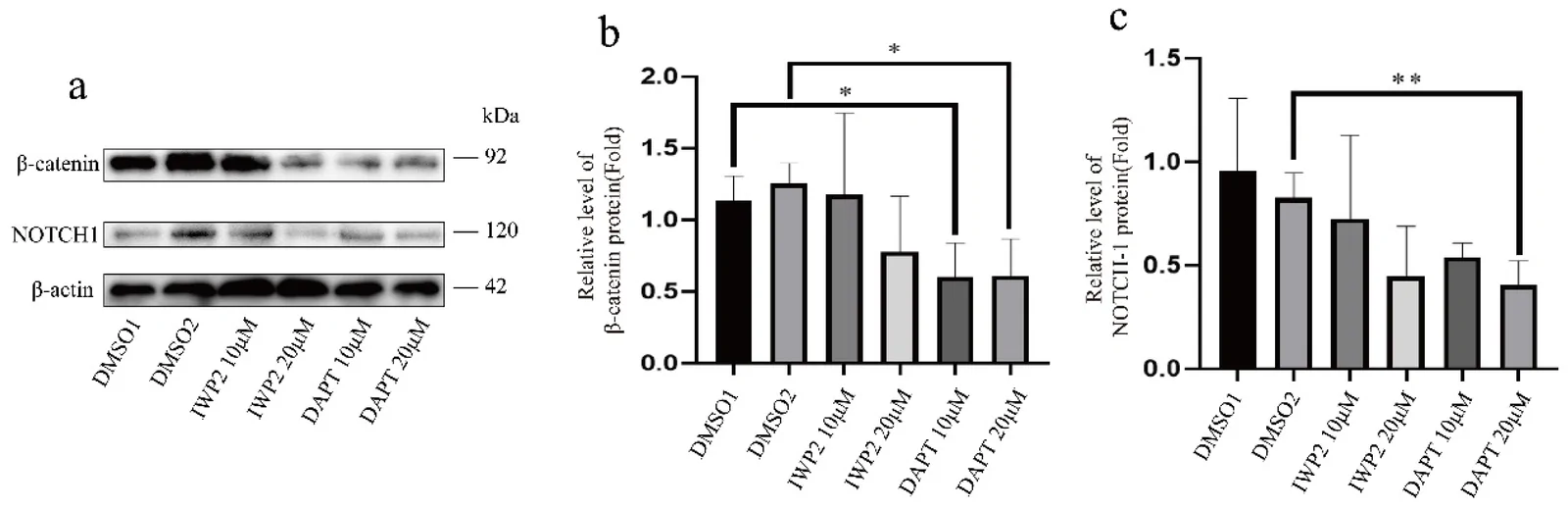
**Crosstalk between Wnt/β-catenin and Notch signaling pathways in IFN-γ-treated** **Ct-infected cells.** (**a**) Western blot analysis of β-catenin and Notch-1 in infected HeLa cells treated with DMSO vehicle, IWP2 (10 or 20 μM), or DAPT (10 or 20 μM). (**b**,**c**) Densitometric quantification of β-catenin and Notch-1, respectively, normalized to β-actin. Data are presented as the mean ± SD from three independent biological experiments. Statistical significance was assessed by one-way ANOVA followed by Dunnett’s multiple-comparison test. * *p* < 0.05, and ** *p* < 0.005.

To examine the relationship between Wnt/β-catenin and Notch signaling under the same continuous IFN-γ treatment condition, infected HeLa cells were treated with the Wnt inhibitor IWP2 or the Notch inhibitor DAPT at 10 and 20 μM and both pathways were analyzed by Western blot. Inhibiting Wnt signaling with IWP2 reduced Notch-1 expression in a dose-dependent manner (*p* < 0.05) (Figure 2a,c). Conversely, blocking Notch signaling with DAPT reduced β-catenin expression (*p* < 0.05) (Figure 2a,b). These reciprocal responses to IWP2 and DAPT were consistent with functional crosstalk between Wnt/β-catenin and Notch signaling and prompted us to further investigate the molecular basis of this interaction.

### 3.3. JAG1 Links Wnt/β-Catenin Signaling to Notch Activation

**Figure 3:**
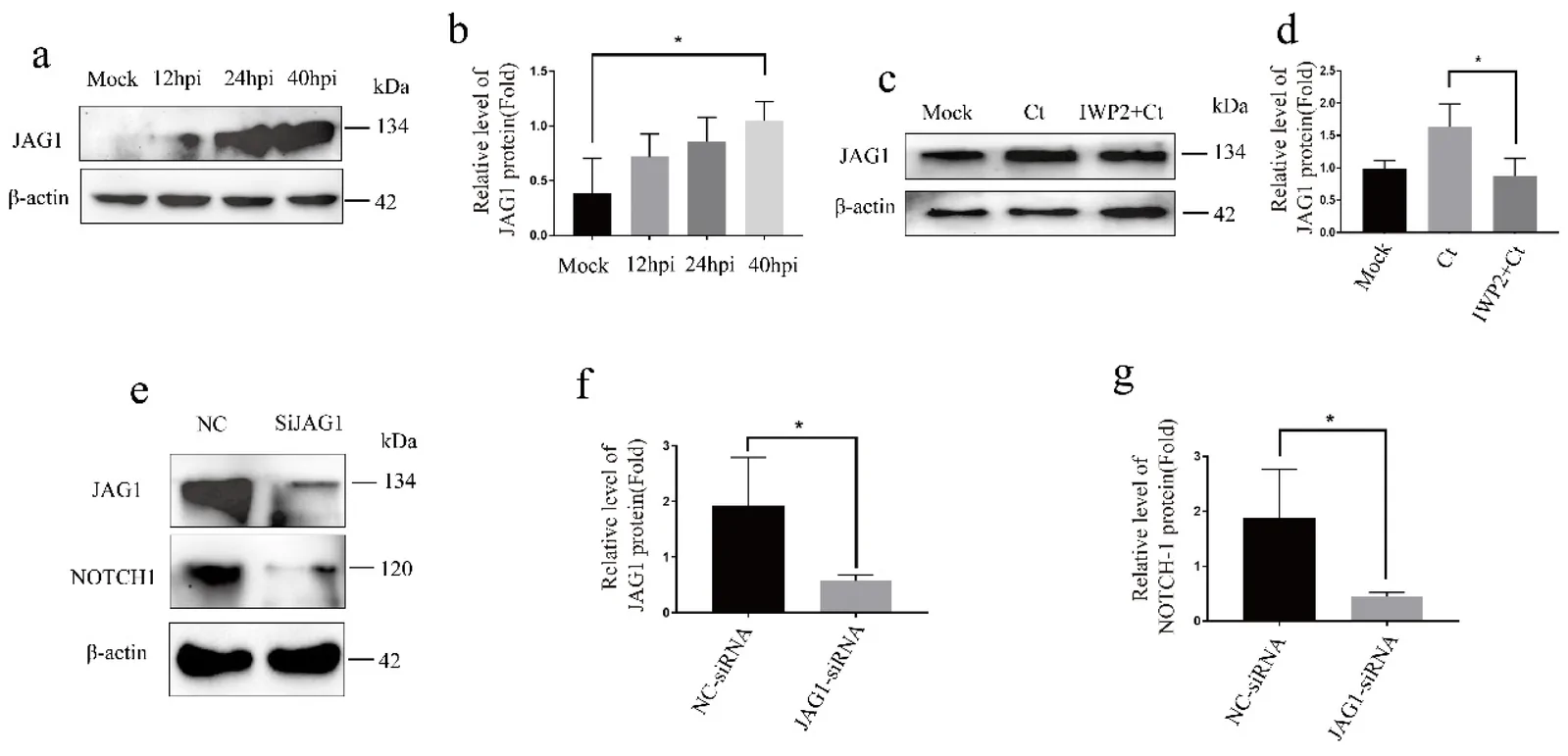
**JAG1 links Wnt/β-catenin signaling to Notch activation in IFN-γ-treated** **Ct-infected cells.** (**a**) The expression of JAG1 in HeLa cells in mock, 12 hpi, 24 hpi, and 40 hpi group. (**b**) Statistics of the relative gray value indicating JAG1 protein expression in panel (**a**). (**c**) The expression of JAG1 in HeLa cells after infection with and without IWP2. (**d**) Statistics of the relative gray value indicating JAG1 protein expression in panel (**c**), quantitative data are presented as the mean ± SD from 3 independent biological experiments. Statistical significance was assessed using one-way ANOVA. * *p* < 0.05. (**e**) The expression of JAG1 and Notch-1 in HeLa cells after Ct infection with JAG1-siRNA. (**f**,**g**) Statistics of the relative gray value indicating JAG1 and Notch-1 protein expression in panel (**e**), quantitative data are presented as the mean ± SD from 3 independent biological experiments. Statistical significance was assessed using *t* tests. * *p* < 0.05.

Given that Wnt/β-catenin and Notch signaling mutually regulate each other, we sought to identify the molecular link between them. JAG1, a Notch ligand and known transcriptional target of β-catenin, was a strong candidate [19]. We first examined JAG1 expression in Ct-infected HeLa cells maintained under continuous IFN-γ treatment. JAG1 levels were significantly higher than in the mock group (*p* < 0.05) (Figure 3a,b). To determine whether Wnt/β-catenin signaling contributes to JAG1 upregulation under this condition, we treated infected cells with IWP2. JAG1 expression dropped significantly following IWP2 treatment (*p* < 0.05) (Figure 3c,d). We then asked whether JAG1 in turn activates Notch signaling. HeLa cells were transfected with JAG1-specific siRNA prior to infection. Knockdown efficiency was confirmed at 70.12% (*p* < 0.05) (Figure 3e,f). JAG1 silencing was accompanied by a significant reduction in Notch-1 expression (*p* < 0.05) (Figure 3e,g), in line with a role for JAG1 in supporting Notch pathway activation. Together, these findings suggest that JAG1 may link Wnt/β-catenin signaling to Notch activation.

### 3.4. Wnt/β-Catenin and Notch Signaling Inhibition Attenuates EMT Phenotypes

**Figure 4:**
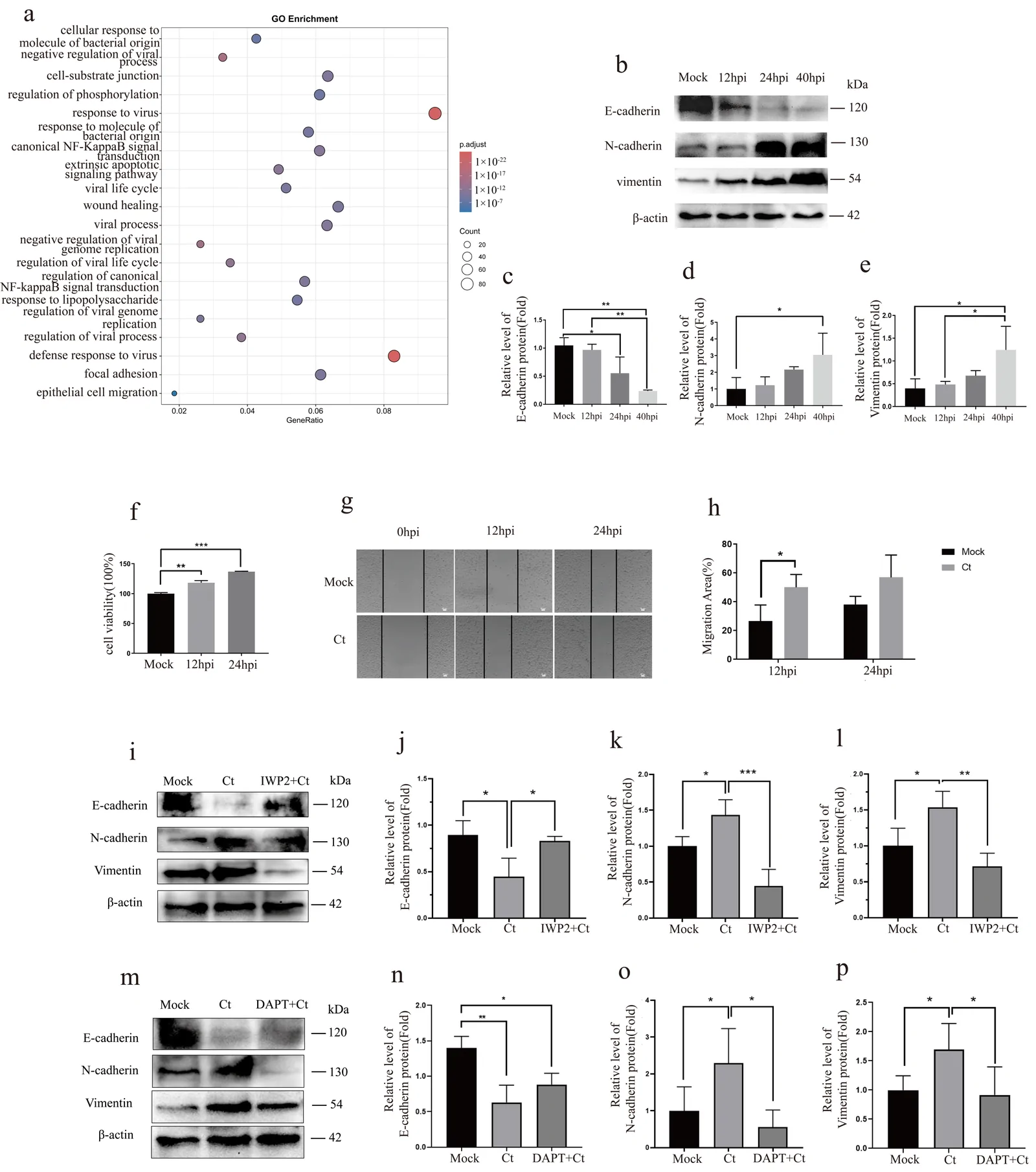
**Wnt/β-catenin–Notch crosstalk promotes an EMT phenotype in IFN-γ-treated** **Ct-infected cells.** (**a**) Bubble diagrams for GO enrichment analysis. (**b**) Expression of EMT-related proteins in mock, 12 hpi, 24 hpi, and 40 hpi group after infection. (**c**–**e**) Quantification of Vimentin, E-cadherin, and N-cadherin expression (gray value ratio of each band to the gray value of β-actin) in panel (**b**), quantitative data are presented as the mean ± SD from 3 independent biological experiments. Statistical significance was assessed using one-way ANOVA. * *p* < 0.05; ** *p* < 0.005. (**f**) Proliferation assay of HeLa cells in mock, 12 hpi, 24 hpi group under the Ct infection model via CCK-8 detection. (**g**,**h**) Microscopic examination diagram of HeLa cells in each group at 0, 12, and 24 h after infection by wound healing assay. Quantification of the migrated cell area at 12 and 24 h after Ct infection. All data represents the results of three independent experiments; statistical significance was assessed using one-way ANOVA. * *p* < 0.05, ** *p* < 0.005, *** *p* < 0.0005. (**i**–**l**) Western blot analysis of EMT-related protein expression was conducted in the Ct infection model using HeLa cells, following treatment with the Wnt/β-Catenin pathway inhibitor IWP2 (10 μM). (**m**–**p**) Western blotting was used to evaluate the expression of EMT-related proteins in HeLa cells using the Ct infection model with the addition of DAPT (10 μM), a Notch pathway inhibitor. Quantitative data are presented as the mean ± SD from 3 independent biological experiments. Statistical significance was assessed using one-way ANOVA. * *p* < 0.05; ** *p* < 0.005. Scale bar in (**g**) = 50 μm.

To determine whether crosstalk between Wnt/β-catenin and Notch signaling contributes to EMT-associated changes, we first examined EMT-related phenotypes. Our previous transcriptomic analysis identified EMT-related biological processes among the significantly enriched GO terms maintained under continuous IFN-γ treatment (Figure 4a). Consistent with this, Western blot confirmed reduced E-cadherin and elevated vimentin and N-cadherin compared to the mock group (Figure 4b–e). Because EMT can be accompanied by changes in proliferative and migratory behavior, we next assessed CCK-8 activity and wound closure at 12 and 24 h post-infection. The CCK-8 signal in Ct-infected cells was 18.07% higher than in the mock group at 12 h (*p* < 0.05) and 36.69% higher at 24 h (*p* < 0.05) (Figure 4f). Wound healing assays showed that the wound-closure rate of the Ct group (50.05%) exceeded that of the mock group (26.58%) at 12 h (*p* < 0.05) and remained higher at 24 h (Ct: 56.99% vs. mock: 38.21%) (Figure 4g,h). We then asked whether inhibition of either pathway attenuated the EMT-related marker changes. Treatment with IWP2 restored E-cadherin expression and reduced both vimentin and N-cadherin levels compared to the infected group (*p* < 0.05) (Figure 4i–l), and DAPT treatment produced the same pattern (*p* < 0.05) (Figure 4m–p). Together, these results indicate that Wnt/β-catenin–Notch crosstalk promotes an EMT phenotype during Ct infection in the presence of IFN-γ.

### 3.5. Wnt/β-Catenin or Notch Inhibition Attenuates EMT and Reduces Infectious Ct Progeny Production

**Figure 5:**
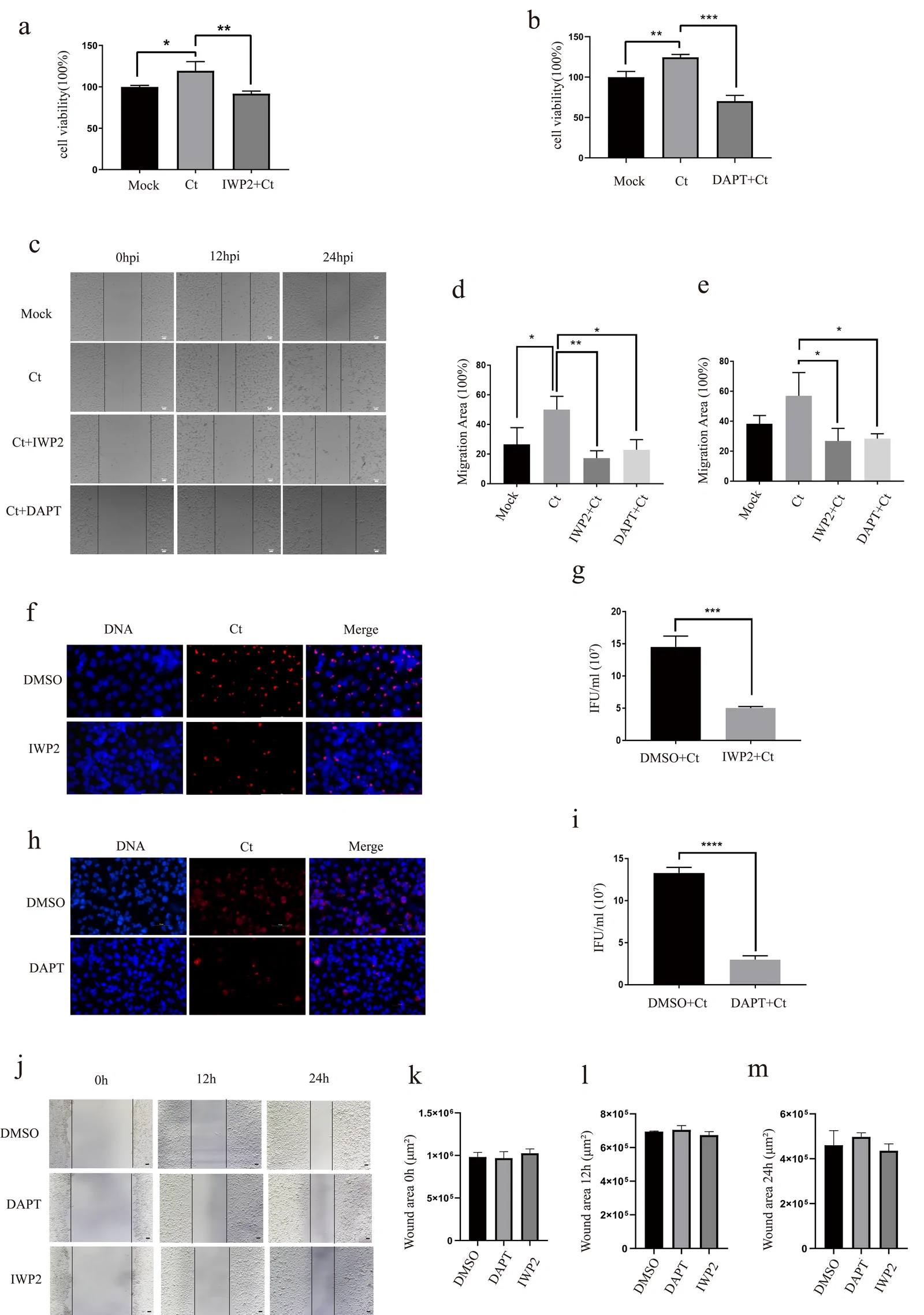
**Wnt/β-catenin or Notch inhibition attenuates EMT and reduces** **Ct** **progeny production.** (**a**) Proliferation assay of HeLa cells in mock, Ct infection group, and IWP2+Ct group via CCK-8 detection. (**b**) HeLa cell proliferation experiment using CCK-8 detection in the Ct infection model in the mock group, Ct infection group, and DAPT + Ct group. (**c**) Microscopic examination diagram of HeLa cells in mock group, Ct infection group, DAPT + Ct group and IWP2 + Ct group by wound healing assay. (**d**) Quantification of the migrated cell area at 12 h in each group. (**e**) Quantification of the migrated cell area at 24 h in each group with IWP2 or DAPT. (**f**,**h**) Representative IFA images of infectious progeny harvested at 40 h from primary cultures treated with DMSO, IWP2 or DAPT and titrated on fresh HeLa monolayers. (**g**,**i**) Corresponding progeny titers (IFU/mL). Nuclei, DAPI (blue); inclusions, Cy3 (red). Data are presented as the mean ± SD; *n* = 3 independent biological experiments. * *p* < 0.05, ** *p* < 0.005, *** *p* < 0.0005, **** *p* < 0.00005. (**j**) Wound-healing assays in uninfected HeLa cells treated with DMSO, DAPT (10 μM), or IWP2 (10 μM), captured at 0, 12, and 24 h. (**k**–**m**) Quantification of wound area at 0, 12, and 24 h, respectively. Data is presented as mean ± SD from at least three independent biological replicates. Statistical significance was assessed by one-way ANOVA. Scale bar in (**c**) = 50 μm.

To assess the functional effects of Wnt/β-catenin or Notch inhibition, we examined host-cell proliferative activity, wound closure, and infectious Ct progeny production under continuous IFN-γ treatment. We assessed cell proliferation using the CCK-8 assay in mock, Ct-infected, and inhibitor-treated groups. The CCK-8 signal in Ct-infected cells was 19.46% higher than in the mock group (*p* < 0.05). IWP2 treatment reduced proliferation by 27.63% relative to the Ct infection group (*p* < 0.05), and DAPT treatment reduced it by 29.73% (*p* < 0.005) (Figure 5a,b). We next examined the effect of these pathways on host cell migration using wound healing assays at 0, 12, and 24 h. At 12 h, the Ct group showed significantly greater wound closure (50.05%) than the IWP2 (17.31%) and DAPT (22.89%) groups (*p* < 0.05) (Figure 5c,d). At 24 h, this pattern was maintained, with the Ct group (56.99%) outperforming the IWP2 (26.83%) and DAPT (28.42%) groups (*p* < 0.05) (Figure 5c,e). To exclude the possibility that reduced migration stemmed from a direct effect of the inhibitors on basal host cell motility, we conducted parallel wound-healing assays in uninfected HeLa cells treated with DMSO, IWP2, or DAPT at the same concentrations. No significant difference in wound closure was detected across groups, indicating that these inhibitors do not suppress basal wound closure in HeLa cells (Figure 5j–m). Together, these findings show that inhibition of Wnt/β-catenin or Notch signaling reduces proliferative activity and wound closure in Ct-infected cells under continuous IFN-γ treatment, consistent with attenuation of EMT-associated phenotypes.

We next examined whether inhibition of Wnt/β-catenin or Notch signaling also affected infectious Ct progeny production under continuous IFN-γ treatment. Ct-infected cells were treated with IWP2, DAPT, or DMSO for 40 h. After treatment, the cells were washed and lysed in fresh medium without IFN-γ or inhibitors. The clarified lysates were then used to infect untreated HeLa cells under standard culture conditions. The infectious progeny titers were determined by IFA and expressed as IFU/mL. In the IWP2-treated group, the infectious progeny titer was (5.038 ± 0.219) × 10^7^ IFU/mL, significantly lower than that of the DMSO control group (14.529 ± 1.660) × 10^7^ IFU/mL (*p* < 0.0005) (Figure 5f,g). Similarly, the infectious progeny titer in the DAPT-treated group was (2.994 ± 0.456) × 10^7^ IFU/mL, compared with (13.288 ± 0.669) × 10^7^ IFU/mL in the DMSO control group (*p* < 0.00005) (Figure 5h,i). Overall, inhibition of Wnt/β-catenin or Notch signaling attenuated EMT-associated changes and reduced infectious Ct progeny production, supporting roles for both pathways in host-cell remodeling and chlamydial development under this condition.

## 4. Discussion

EMT is a multifaceted process that alters epithelial identity and is often accompanied by changes in cell proliferation and motility [20]. In our experimental setting, infected cells maintained under continuous IFN-γ treatment showed higher CCK-8 signals than the mock group, consistent with enhanced proliferative activity (Figure 4f). Previous studies have shown that Ct infection can promote host-cell proliferative responses and cell-cycle re-entry in a context-dependent manner [21,22]. Infected cells also showed faster wound closure at both time points (Figure 4g). These cellular responses were accompanied by increased vimentin and N-cadherin expression and decreased E-cadherin expression (Figure 4b). Thus, Ct infection under continuous IFN-γ treatment was accompanied by molecular and cellular features associated with EMT.

Previous studies have implicated Wnt/β-catenin and Notch signaling in chlamydia-associated epithelial remodeling. Kessler et al. reported dysregulation of Wnt signaling in infected fallopian tube epithelium [23]. More recently, single-cell transcriptomic analysis of Ct-infected cervical organoids revealed enrichment and infection-associated rewiring of Notch signaling in ectocervical epithelial populations [24]. Altered β-catenin localization, including disruption of N-cadherin/β-catenin junctional complexes and β-catenin redistribution, has also been observed in infected cervical epithelial cells [25]. However, Wnt/β-catenin and Notch signaling have largely been examined separately and at a descriptive or correlative level, leaving their functional interaction during Ct infection and its influence on EMT unresolved. Extending these observations, we found that Ct infection activated both Wnt/β-catenin and Notch signaling in HeLa cells maintained under continuous IFN-γ treatment. Inhibition of either pathway reduced activation of the other and attenuated EMT-associated phenotypes. Together, these findings support a reciprocal functional interaction between Wnt/β-catenin and Notch signaling, with JAG1 as a potential link, and suggest that this interaction promotes EMT in this setting.

The EMT-regulatory functions of Wnt/β-catenin and Notch signaling have been demonstrated in several pathological settings. Wnt/β-catenin activation is associated with E-cadherin loss and increased expression of N-cadherin and vimentin [26], whereas activation of Notch1 can promote EMT through transcriptional reprogramming [27]. Functional interactions between Wnt/β-catenin and Notch signaling may be cooperative or antagonistic depending on the cellular context [28,29]. Under continuous IFN-γ treatment, IWP2 reduced Notch-related readouts, whereas DAPT decreased β-catenin levels (Figure 2a–c), supporting reciprocal regulation between the two pathways. The corresponding attenuation of EMT marker changes following either treatment further supports positive functional interplay between Wnt/β-catenin and Notch signaling. Previous studies have similarly shown that modulation of host Wnt/β-catenin signaling affects Ct development and EB production [30], consistent with the reduced infectious progeny observed following IWP2 treatment in our study.

**Figure 6:**
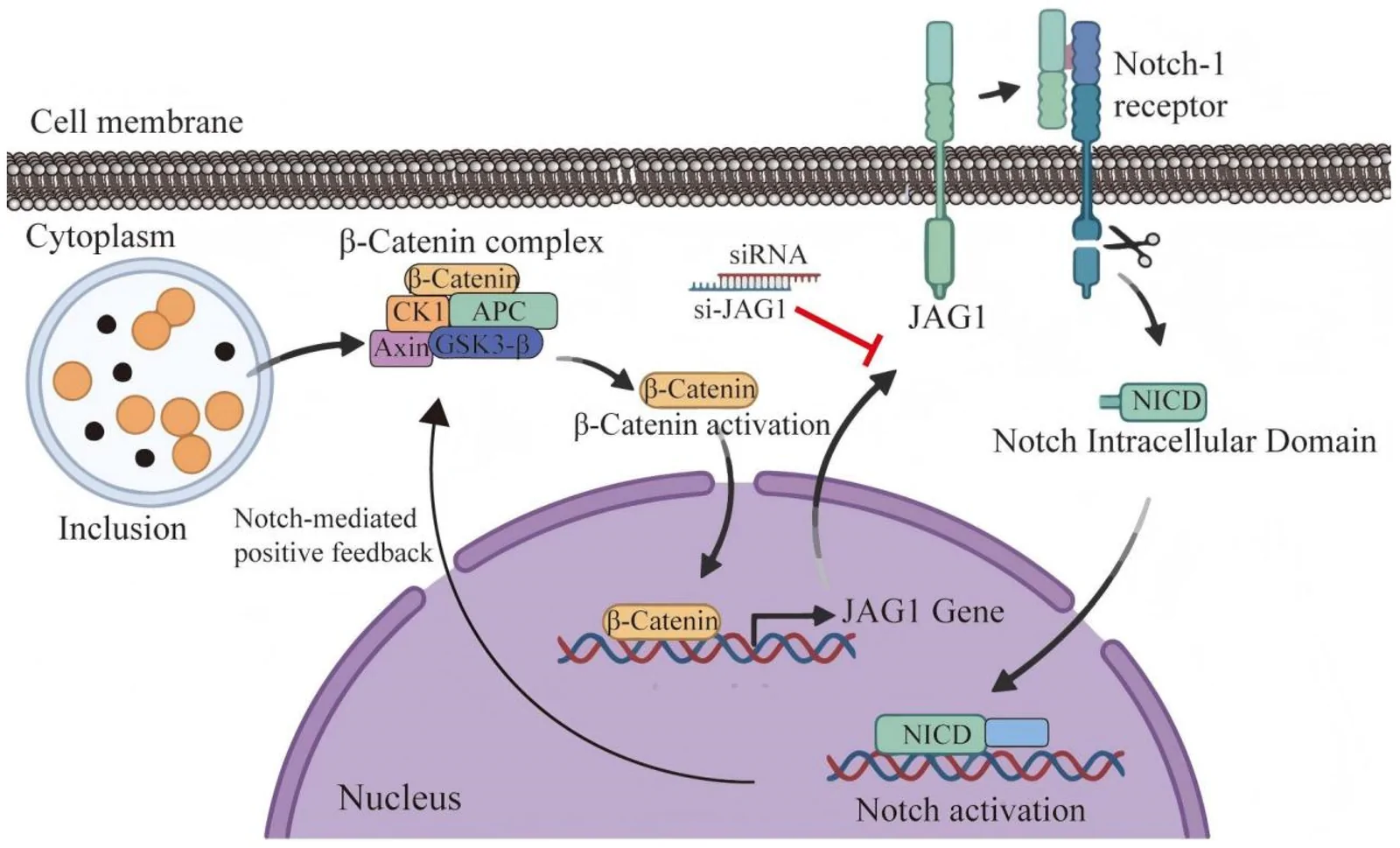
**Proposed model of JAG1-mediated Wnt/β-catenin–Notch crosstalk inducing EMT during** **Ct** **infection.** During Ct infection, Wnt/β-catenin signaling is activated and β-catenin translocates to the nucleus to induce JAG1 expression. JAG1 then promotes Notch1 activation and NICD nuclear translocation, establishing a Wnt/β-catenin–Notch crosstalk that promotes EMT. JAG1 knockdown suppresses these effects.

JAG1 provides a potential molecular link between Wnt/β-catenin and Notch signaling. As a canonical Notch ligand, JAG1 activates downstream signaling and regulates cell fate, proliferation, and differentiation [31]. In Ct-infected cells under continuous IFN-γ treatment, JAG1 expression increased (Figure 3a,b), whereas IWP2 treatment reduced its expression (Figure 3c,d). Moreover, JAG1 silencing significantly decreased Notch1 expression (Figure 3e–g). Together with the reciprocal pathway responses to IWP2 and DAPT, these findings support JAG1 as a potential contributor to Wnt/β-catenin–Notch crosstalk under this treatment condition. We therefore propose a model in which β-catenin-associated JAG1 upregulation enhances Notch signaling and contributes to EMT-related changes (Figure 6).

Beyond their effects on EMT, inhibition of Wnt/β-catenin or Notch signaling also reduced infectious Ct progeny production under continuous IFN-γ treatment (Figure 5f–i). Together with the attenuation of EMT-associated phenotypes, these findings link both pathways to host-cell remodeling and chlamydial intracellular development. These parallel changes raise the possibility that EMT-associated host-cell remodeling may favor the intracellular development of Ct.

One limitation of this study is the use of HeLa cells, which do not fully reproduce the biological complexity of human reproductive epithelium. Confirmation in primary reproductive tract epithelial cells will be important to determine whether the same Wnt/Notch–EMT relationship occurs in a more physiologically relevant model.

## 5. Conclusions

In conclusion, this study identifies functional crosstalk between Wnt/β-catenin and Notch signaling, potentially involving JAG1, that contributes to EMT-associated changes in Ct-infected cells under continuous IFN-γ treatment. Inhibition of either pathway attenuated these changes and was associated with reduced infectious progeny production.

## Figures and Tables

**Table 1:** Primer sequence.

Genes	Forward	Reverse
GAPDH	ACAGCCTCAAGATCATCAGC	GGTCATGAGTCCTTCCACGAT
Notch-1	TGGAAGCTACTGACTTAGTAGGGGGAAAAC	GCAAGCATGAAGTGGTCCAGGGTGTGAGTG GTGTGAGTG
Hes1	TGGAGAAGAGGCGAAGG	CGGAGGTGCTTCACAGTC

## Data Availability

The raw data of the RNA-seq data files were submitted to the Sequence Read Archive (SRA) database under the BioProject accession number PRJNA1036572 (https://www.ncbi.nlm.nih.gov/bioproject/PRJNA1036572, accessed on 5 March 2025).

## References

[1] Truong H.-H.M., Otieno B., Kadede K., Odeny D., Opiyo M., Hewa M., Opondo F., Heylen E., Amboka S., Odhiambo H. (2025). Prevalence of chlamydia and gonorrhoea among Kenyan adolescents. Sex. Transm. Infect..

[2] Hocking J.S., Geisler W.M., Kong F.Y.S. (2023). Update on the Epidemiology, Screening, and Management of *Chlamydia trachomatis* Infection. Infect. Dis. Clin. N. Am..

[3] Darville T. (2021). Pelvic Inflammatory Disease Due to *Neisseria gonorrhoeae* and *Chlamydia trachomatis*: Immune Evasion Mechanisms and Pathogenic Disease Pathways. J. Infect. Dis..

[4] Alexiou Z.W., Hoenderboom B.M., Hoebe C.J.P.A., Dukers-Muijrers N.H.T.M., Götz H.M., Van Der Sande M.A.B., De Vries H.J.C., Den Hartog J.E., Morré S.A., Van Benthem B.H.B. (2024). Reproductive tract complication risks following *Chlamydia trachomatis* infections: A long-term prospective cohort study from 2008 to 2022. Lancet Reg. Health-Eur..

[5] Horner P.J., Flanagan H., Horne A.W. (2021). Is There a Hidden Burden of Disease as a Result of Epigenetic Epithelial-to-Mesenchymal Transition Following *Chlamydia trachomatis* Genital Tract Infection?. J. Infect. Dis..

[6] Caven L.T., Carabeo R.A. (2023). The role of infected epithelial cells in Chlamydia-associated fibrosis. Front. Cell. Infect. Microbiol..

[7] Ling H., Luo L., Dai X., Chen H. (2022). Fallopian tubal infertility: The result of Chlamydia trachomatis-induced fallopian tubal fibrosis. Mol. Cell. Biochem..

[8] Zhang C.X., Huang R.Y.-J., Sheng G., Thiery J.P. (2025). Epithelial-mesenchymal transition. Cell.

[9] Zadora P.K., Chumduri C., Imami K., Berger H., Mi Y., Selbach M., Meyer T.F., Gurumurthy R.K. (2019). Integrated Phosphoproteome and Transcriptome Analysis Reveals Chlamydia-Induced Epithelial-to-Mesenchymal Transition in Host Cells. Cell Rep..

[10] Caven L.T., Brinkworth A.J., Carabeo R.A. (2023). *Chlamydia trachomatis* induces the transcriptional activity of host YAP in a Hippo-independent fashion. Front. Cell. Infect. Microbiol..

[11] Darville T., Sun X., Zhang Y., O’Connell C.M., Mokashi N.V., Tang W., Bhardwaj A., Duncan B., Andrews C.W., Wiesenfeld H.C. (2026). miRNA/mRNA analysis of increased TGF-β pathways drive epithelial-mesenchymal transition and regulatory T cell differentiation. Front. Immunol..

[12] Caven L., Carabeo R. (2023). Chlamydial YAP activation in host endocervical epithelial cells mediates pro-fibrotic paracrine stimulation of fibroblasts. mSystems.

[13] Liu J., Xiao Q., Xiao J., Niu C., Li Y., Zhang X., Zhou Z., Shu G., Yin G. (2022). Wnt/β-catenin signalling: Function, biological mechanisms, and therapeutic opportunities. Signal Transduct. Target. Ther..

[14] Zhou B., Lin W., Long Y., Yang Y., Zhang H., Wu K., Chu Q. (2022). Notch signaling pathway: Architecture, disease, and therapeutics. Signal Transduct. Target. Ther..

[15] Wen Y., Luo F., Zhao L., Su S., Lei W., Liu Y., Shi K., Li Z. (2021). Long Non-Coding RNA FGD5-AS1 Induced by *Chlamydia trachomatis* Infection Inhibits Apoptosis via Wnt/β-Catenin Signaling Pathway. Front. Cell. Infect. Microbiol..

[16] Xiao K., Liu C., Wang H., Hou F., Shi Y., Qian Z.R., Zhang H., Deng D.Y.B., Xie L. (2023). Umbilical cord mesenchymal stem cells overexpressing CXCR7 facilitate treatment of ARDS-associated pulmonary fibrosis via inhibition of Notch/Jag1 mediated by the Wnt/β-catenin pathway. Biomed. Pharmacother..

[17] Luo F., Wen Y., Zhao L., Su S., Lei W., Chen L., Chen C., Huang Q., Li Z. (2022). LncRNA ZEB1-AS1/miR-1224-5p/MAP4K4 axis regulates mitochondria-mediated HeLa cell apoptosis in persistent *Chlamydia trachomatis* infection. Virulence.

[18] Singh A., Zapata M., Sung Choi Y., Yoon S.-O. (2014). GSI promotes vincristine-induced apoptosis by enhancing multi-polar spindle formation. Cell Cycle.

[19] Song P., Gao Z., Bao Y., Chen L., Huang Y., Liu Y., Dong Q., Wei X. (2024). Wnt/β-catenin signaling pathway in carcinogenesis and cancer therapy. J. Hematol. Oncol..

[20] Serresi M., Kertalli S., Li L., Schmitt M.J., Dramaretska Y., Wierikx J., Hulsman D., Gargiulo G. (2021). Functional antagonism of chromatin modulators regulates epithelial-mesenchymal transition. Sci. Adv..

[21] Koster S., Gurumurthy R.K., Kumar N., Prakash P.G., Dhanraj J., Bayer S., Berger H., Kurian S.M., Drabkina M., Mollenkopf H.-J. (2022). Modelling Chlamydia and HPV co-infection in patient-derived ectocervix organoids reveals distinct cellular reprogramming. Nat. Commun..

[22] Ekka R., Gutierrez A., Johnson K.A., Tan M., Sütterlin C. (2024). *Chlamydia trachomatis* induces disassembly of the primary cilium to promote the intracellular infection. PLoS Pathog..

[23] Kessler M., Zielecki J., Thieck O., Mollenkopf H.-J., Fotopoulou C., Meyer T.F. (2012). *Chlamydia trachomatis* Disturbs Epithelial Tissue Homeostasis in Fallopian Tubes via Paracrine Wnt Signaling. Am. J. Pathol..

[24] Prakash P.G., Kumar N., Koster S., Wentland C., Dhanraj J., Gurumuthy R.K., Chumduri C. (2025). Single-cell atlas of cervical organoids uncovers epithelial immune heterogeneity and intercellular cross-talk during Chlamydia infection. Sci. Adv..

[25] Prozialeck W.C., Fay M.J., Lamar P.C., Pearson C.A., Sigar I., Ramsey K.H. (2002). *Chlamydia trachomatis* Disrupts N-Cadherin-Dependent Cell-Cell Junctions and Sequesters β-Catenin in Human Cervical Epithelial Cells. Infect. Immun..

[26] Gorka J., Marona P., Kwapisz O., Waligórska A., Pospiech E., Dobrucki J.W., Rys J., Jura J., Miekus K. (2021). MCPIP1 inhibits Wnt/β-catenin signaling pathway activity and modulates epithelial-mesenchymal transition during clear cell renal cell carcinoma progression by targeting miRNAs. Oncogene.

[27] Saran U., Chandrasekaran B., Tyagi A., Shukla V., Singh A., Sharma A.K., Damodaran C. (2023). A small molecule inhibitor of Notch1 modulates stemness and suppresses breast cancer cell growth. Front. Pharmacol..

[28] Turetti F., Dokoupil M., Collu G.M., Harnos J., Mašek J. (2026). Decoding ‘Wntch’: The intertwined Wnt and Notch pathways in development and disease. Open Biol..

[29] Hu L., Chen W., Qian A., Li Y.-P. (2024). Wnt/β-catenin signaling components and mechanisms in bone formation, homeostasis, and disease. Bone Res..

[30] Kintner J., Moore C.G., Whittimore J.D., Butler M., Hall J.V. (2017). Inhibition of Wnt Signaling Pathways Impairs *Chlamydia trachomatis* Infection in Endometrial Epithelial Cells. Front. Cell. Infect. Microbiol..

[31] Li X.-J., Morgan C., Li L., Zhang W.-Y., Chrysostomou E., Doetzlhofer A. (2025). The Notch ligand Jagged1 plays a dual role in cochlear hair cell regeneration. Nat. Commun..

